# Successive Fermentation of Aguamiel and Molasses by *Aspergillus oryzae* and *Saccharomyces cerevisiae* to Obtain High Purity Fructooligosaccharides

**DOI:** 10.3390/foods11121786

**Published:** 2022-06-17

**Authors:** Orlando de la Rosa, Adriana Carolina Flores-Gallegos, Diana Muñíz-Márquez, Juan C. Contreras-Esquivel, José A. Teixeira, Clarisse Nobre, Cristóbal N. Aguilar

**Affiliations:** 1 Bioprocesses & Bioproducts Research Group, Food Research Department, School of Chemistry, Universidad Autónoma de Coahuila, Saltillo 25280, Mexico; orlandorosaflores@uadec.edu.mx (O.d.l.R.); carolinaflores@uadec.edu.mx (A.C.F.-G.); carlos.contreras@uadec.edu.mx (J.C.C.-E.); 2 Tecnológico Nacional de México, Instituto Tecnológico de Ciudad Valles, Ciudad Valles 79010, Mexico; diana.marquez@tecvalles.mx; 3 Centre of Biological Engineering, Campus de Gualtar, University of Minho, 4710-057 Braga, Portugal; jateixeira@deb.uminho.pt; 4 LABBELS—Associate Laboratory, 4710-057 Braga, Portugal

**Keywords:** *Aspergillus oryzae*, whole-cell fermentation, fructooligosaccharides, low-cost media, aguamiel, molasses, successive fermentation

## Abstract

Fructooligosaccharides (FOS) are usually synthesized with pure enzymes using highly concentrated sucrose solutions. In this work, low-cost aguamiel and molasses were explored as sucrose alternatives to produce FOS, via whole-cell fermentation, with an *Aspergillus oryzae* DIA-MF strain. FOS production process was optimized through a central composite experimental design, with two independent variables: initial sucrose concentration in a medium composed of aguamiel and molasses (AgMe), and inoculum concentration. The optimized process—165 g/L initial sucrose in AgMe (adjusted with concentrated molasses) and 1 × 10^7^ spores/mL inoculum concentration—resulted in an FOS production of 119 ± 12 g/L and a yield of 0.64 ± 0.05 g _FOS_/g _GFi_. Among the FOSs produced were kestose, nystose, 1-fructofuranosyl-nystose, and potentially a novel trisaccharide produced by this strain. To reduce the content of mono- and disaccharides in the mixture, run a successive fermentation was run with two *Saccharomyces cerevisiae* strains. Fermentations run with *S. cerevisiae* S227 improved FOS purity in the mixture from 39 ± 3% to 61.0 ± 0.6% (*w*/*w*) after 16 h of fermentation. This study showed that agro-industrial wastes such as molasses with aguamiel are excellent alternatives as substrate sources for the production of prebiotic FOS, resulting in a lower-cost process.

## 1. Introduction

Fructooligosaccharides (FOS) are well documented in pharmaceutical and biotechnological industries due to their broad scope of health applications derived from their prebiotic activity [1,2,3]. FOS are sweet non-cariogenic carbohydrates and water-soluble non-digestible fibers, which are frequently included in low-calorie diets [4,5,6]. FOS fermentation by gut microbiota allows for a series of benefits such as the regulation of gut microbial populations, improved resistance against pathogenic bacteria, improved overall gut health by regulation of the immune response, the activation of lymphocytes and phagocytes, cell proliferation and differentiation, and enhanced nutrient absorption, all linked to the short-chain fatty acids generated by probiotic bacteria [7,8,9,10].

FOSs are carbohydrates from the fructan family composed of a sucrose molecule linked to a series of fructose units bonded by a linear (β2→1)-glycosidic bond in the case of inulin-type FOS found in plants and synthesized by fungi. Levan-type fructans have (β2→6)-linked fructose residues and are found in plants and mainly synthesized by levansucrase from bacteria and some fungi [11], while the branched group neo-series are composed of a mix of (β2→1) and (β2→6)-glycosidic bonds (e.g., graminan) [12].

Industrially, inulin-type FOSs can be produced either by inulin hydrolysis or by synthesis using transfructosylation enzymes such as β-fructofuranosidase (FFases, EC 3.2.1.26) and fructosyltransferase (FTases, EC 2.4.1.9) that catalyze sucrose bioconversion (Figure S1). These enzymes are present in plants, bacteria, yeast, and fungal sources [13,14,15,16,17,18]. Among the fungal sources, strains from the genera *Aspergillus* have proven relevant for inulin-type FOS synthesis [18,19,20,21,22]. Enzymes can be produced by fermentation in a medium rich in sucrose. Furthermore, several enzyme purification and concentration steps are required before a second fermentation, where FOSs are finally produced in a high-concentration sucrose solution. Whole-cell fermentation is a recently explored strategy for FOS production [18,23,24,25,26]. It is a process where the enzyme and FOSs are synthesized in a single fermentation, which avoids the enzyme purification step. The use of the whole-cell process has advantages such as cost reduction, process simplicity, and broader operation parameters because of the better cell stability under different process conditions [18,23,24,27,28].

When enzymes are produced for industrial purposes, around 30 to 40% of the production cost is used in the microorganism growth medium, mainly constituted by carbon and nitrogen sources [29]. Addressing that challenge, alternative carbon sources equivalent to commercially available sucrose have been investigated [30,31,32,33,34,35]. 

Aguamiel is a cheap fermentable product from the *Agave* plant used for the traditional fermented beverage called “pulque.” It contains sucrose, fructose, glucose, and FOS [36]. Molasses are a by-product obtained from the industrial process of sucrose production [37]. Molasses have been widely recognized as a low-cost raw material for the production of oligosaccharides, polysaccharides, and other functional sugars [38,39,40]. The use of alternative sucrose sources as culture media, such as aguamiel or some industrial sugar refinery intermediate processing streams, such as molasses, may reduce the production costs of the process as compared with a culture medium enriched with commercial sucrose [34,41,42]. Moreover, it adds value to the agro-industrial by-products.

FOS mixtures obtained by fermentation have a high number of monosaccharides and disaccharides in their composition. Given that FOSs’ beneficial effects could be hindered by the presence of these saccharides, several strategies have been reported for their removal. Among them is the successive fermentation, which holds high development potential [24,43,44,45,46,47]. In this strategy, a second fermentation is performed by a microorganism (other than the FOS producer) to deplete the small saccharides from the resulting FOS mixture [24,25,28]. This work aimed to develop a low-cost process for FOS production using aguamiel and molasses as alternative sucrose sources and evaluate successive fermentation strategies to obtain FOS at high purity.

## 2. Materials and Methods

### 2.1. Raw Material Collection and Treatment

Aguamiel from maguey (*Agave salmiana*) was collected in Las Mangas locality in Saltillo, Coahuila, Mexico. The Aguamiel was filtered, distributed in batches, and stored in the freezer until its use. The sugarcane molasses was provided by the sugar mill “El ingenio de San Luis” located in Ciudad Valles, San Luis Potosí, Mexico. It was distributed in batches and stored until its use. Before its use, aguamiel and cane molasses were treated by a thermal process at 121 °C for 15 min, and sugars were determined by high performance liquid chromatography (HPLC) as described in Section 2.6 (Table S1).

### 2.2. Microorganisms

*Aspergillus oryzae* DIA-MF was obtained from the microorganism collection of the Food Research Department of Autonomous University of Coahuila (Saltillo, Mexico). *Saccharomyces cerevisiae* 227 and *Saccharomyces cerevisiae* 200 were provided by microorganism collection of Instituto Tecnológico de Durango (Durango, México). *Aspergillus oryzae* DIA-MF was cultured on potato dextrose agar (PDA) at 30 °C for five days for subsequent spore harvest. *Saccharomyces cerevisiae* strains were pre-cultured on yeast extract peptone dextrose medium (YPD) at 30 °C and 150 rpm of agitation for 48 h. 

### 2.3. Evaluation of Aguamiel/Molasses-Based Media for FOS Production

Different media were evaluated for FOS production: aguamiel, aguamiel and molasses (AgMe), and molasses (Me); sucrose concentrations were set according to the concentration of sugars present in aguamiel and molasses. The media were evaluated by liquid fermentation using *Aspergillus oryzae* DIA-MF (2 × 10^6^ spore/mL). Fermentations were carried out for 48 h, sampling every 8 h; from each sample, biomass was recovered by filtration and determined gravimetrically. The pH and the biomass were monitored during the fermentation; the concentration of sucrose, glucose, fructose, and FOS produced was quantified by HPLC [48]. The best condition was selected according to the best production yield, based on FOS production from initial sucrose (GFi) [g_FOS_/g_GFi_], and productivity. Data were compared using one-way ANOVA followed by a Tukey’s multiple comparison test with a 95% confidence level. Positive effects were considered significant for *p*-values lower than 0.05. The culture media with the highest yield [g_FOS_/g_GFi_] was selected for further optimization. A central composite design 2^2^ (CCD) was applied considering inoculum and sucrose concentration at three levels, basal (0), maximum (+1), and minimum (−1), and having as response variable the FOS yield. The CCD matrix is shown in Table 1.

Fermentation assays were carried out in 125 mL flasks containing 30 mL of AgMe culture medium formulated with different sucrose concentrations according to the treatment. The initial pH of the media in all treatments was adjusted to 5.5, and flasks were incubated in a shaker at 180 rpm at 30 °C for 36 h. Samples were harvested at regular intervals. After optimization, assays were performed to validate the model. All experiments were carried out in triplicate. 

Data analysis was performed using STATISTICA software version 10.0 (Statsoft, Tulsa, OK, USA). The model evaluated the different effects of the independent variables on FOS yield. The quality of the fitted model was verified statistically by the magnitude of the coefficient of determination R^2^, and its statistical significance was evaluated by the F-test analysis of variance (ANOVA). Data comparison was performed using one-way ANOVA and a subsequent Tukey’s multiple comparison test with a 95% confidence level. Positive effects were considered significant for *p*-values less than 0.05.

### 2.4. Bioreactor Trials

After selecting the best conditions for FOS production, the fermentation was scaled up at the bioreactor level. 

#### 2.4.1. Pre-Inoculum

A 100 mL pre-inoculum was prepared in a 250 mL flask with the modified Czapek dox medium (g/L): 200 sucrose, 5.0 NaNO_3_, 4.0 KH_2_PO_4_, 0.5 KCl, 0.35 K_2_SO_4_, 0.5 MgSO_4_·7H_2_O, and 0.01 FeSO_4_·7H_2_O, as reported by Nobre et al. [24]. The medium was sterilized at 121 °C for 15 min and subsequently inoculated with a spore solution of 2 × 10^6^ spores/mL and incubated for 3 days at 30 °C and 180 rpm.

#### 2.4.2. Fermentation

Fermentation was carried out in a bioreactor (Benchtop fermenter type RALF Bioengineering AG, Wald, Switzerland) of 2 L capacity with a working volume of 1 L with the AgMe medium (with the optimized sucrose concentration), which was autoclaved at 121 °C for 30 min. As a control, modified Czapek-Dox was also used to evaluate FOS production by *A. Oryzae* DIA-MF in a defined synthetic medium (g/L): 200 sucrose, 17 yeast extract, 5.0 NaNO_3_, 4.0 KH_2_PO_4_, 0.5 KCl, 0.35 K_2_SO_4_, 0.5 MgSO_4_·7H_2_O, and 0.01 FeSO_4_·7H_2_O [24]. Sucrose and FeSO_4_·7H_2_O solutions were sterilized by filtration (0.2 µm), and the other solutions were sterilized in an autoclave at 121 °C for 15 min. The working conditions of the bioreactor used were 30 °C, 200 rpm, and pH 5.5, which was maintained during fermentation by the controlled addition of ortho-phosphoric acid and ammonium hydroxide solution. Fermentations were evaluated by collecting samples every 6 h to quantify the sugars; quantification was performed by HPLC [48]. The best production time was defined based on the best yield (g_FOS_/g_GFi)_ and purity of FOS (g_FOS_/g_other carbohydrates_) achieved during the fermentation.

### 2.5. Successive Fermentation with Yeast

A successive fermentation was carried out with the yeast *Saccharomyces cerevisiae* to remove the residual sugars from the mixture generated during the production of FOS (glucose, fructose, and sucrose). Two strains of *Saccharomyces cerevisiae* were evaluated: *S. cerevisiae* 227 (S227) and *S. cerevisiae* 200 (S200); strains were activated in YPD medium for 48 h at 30 °C and 150 rpm, and subsequently inoculated (2 × 10^7^ cells/mL) in the FOS mixture obtained from a previous fermentation. Fermentations were carried out in a flask at 30 °C, 150 rpm, with an initial pH of 5.5. Samples were harvested every 8 h for 40 h to monitor the consumption of glucose, fructose, sucrose, and FOS content.

### 2.6. HPLC Analysis

Samples were filtered through 0.22 μm nylon filters (Millipore, St. Louis, MO, USA) and placed in special vials for HPLC. The analysis was performed on a Varian Pro-Star330 HPLC instrument under the following conditions: Carbohydrate Prevail ES column (5 μm, 250 mm × 4.6 mm) at 30 °C using a refractive index (RI) detector at the same temperature. As a mobile phase, an acetonitrile/water solution (70/30 *v*/*v*) with 0.04% NH_4_OH in water was used at a 1.0 mL/min flow. Standard curves were made with known concentrations of fructose (F), glucose (G), and sucrose (GF) (Sigma Aldrich, St. Louis, MO, USA) and FOS 1-kestose (GF_2_), 1-nystose (GF_3_), and 1F-fructofuranosylnystose (GF_4_) (Wako Pure Chemical Industries, Ltd., Osaka, Japan) [48].

### 2.7. Experimental Design and Statistical Analysis

Data were evaluated by analysis of variance (ANOVA) using STATISTICA 10.0 software (Statsoft, Tulsa, OK, USA); when needed, mean treatments were compared using Tukey’s multiple-range procedure. A *p*-value of less than 0.05 was regarded as showing a significant difference.

## 3. Results

### 3.1. FOS Production with Different Aguamiel–Molasses Media

In the fermentations conducted with *Aspergillus oryzae* DIA-MF, good production of FOS and effective conversion of sucrose were obtained independently of the media composition used. To compare the fermentations run with the different culture media and determine the most efficient one for FOS production, fermentation yield (Y = g_FOS_/g_initial sucrose (GFi)_) and purity ((g_FOS_/g_Total sugars_) × 100) were calculated (Table 2). The best results were achieved with the AgMe media. A maximum concentration of 46.7 ± 0.3 g/L of FOS (28.80 ± 0.17 g/L of GF_2_ and 17.93 ± 0.14 g/L of GF_3_) with the best FOS production yield (0.61 g ± 0.06 g_FOS_/g_GFi_) was achieved. No significant differences were found with the Me media, but the same yield was achieved at a lower fermentation time, 16 h instead of 24 h, and with higher FOS purity (39.99 ± 0.26%). Thus, AgMe was selected as the best culture medium for FOS production and was used in the optimization assays further discussed.

### 3.2. FOS Production Optimization

As sucrose concentration has been reported as the main factor affecting FOS production by transfructosylation, different concentrations of initial sucrose (GFi) (100, 150, and 200 g/L) in the AgMe medium were considered for optimization. The effect of the inoculum concentration was also studied, and three inoculum concentrations were tested (1 × 10^6^, 1 × 10^7^, and 1 × 10^8^ sp/mL). 

**Figure 1:**
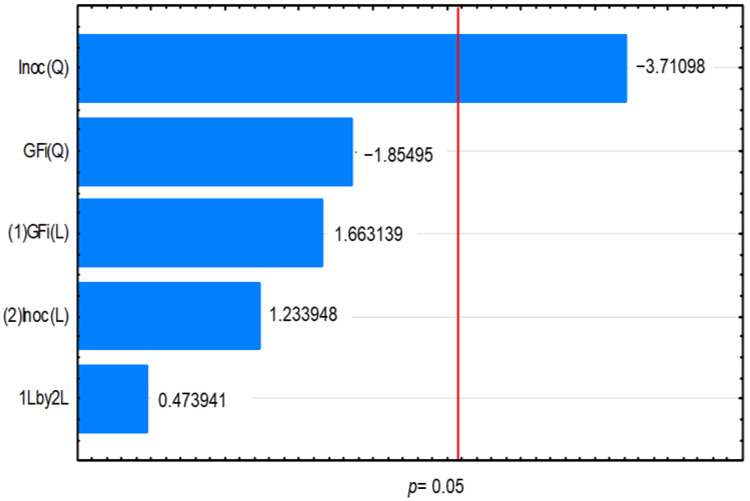
Pareto chart of the standardized effects of the variables initial sucrose (GFi) and inoculum concentration (Inoc) on the fructooligosaccharides production yield.

The only statistically significant factor in the production yield was given by the quadratic effect of the inoculum concentration (Figure 1). High values of inoculum concentration reduced FOS production yield, which can be related to a major requirement of sucrose for cells’ metabolic demand, leading to less sucrose converted to FOS. Initial sucrose concentration did not show a significant effect on FOS yield. The initial sucrose concentration affects the time when the FOS maximum is reached, and the composition of the mixture of FOS formed, promoting more GF_2_ in some cases or promoting more GF_3_ and GF_4_. However, the sum of the total FOSs per amount of initial sucrose seems to not be affected. The experimental data and predicted results obtained for the CCD are given in Table 3.

**Figure 2:**
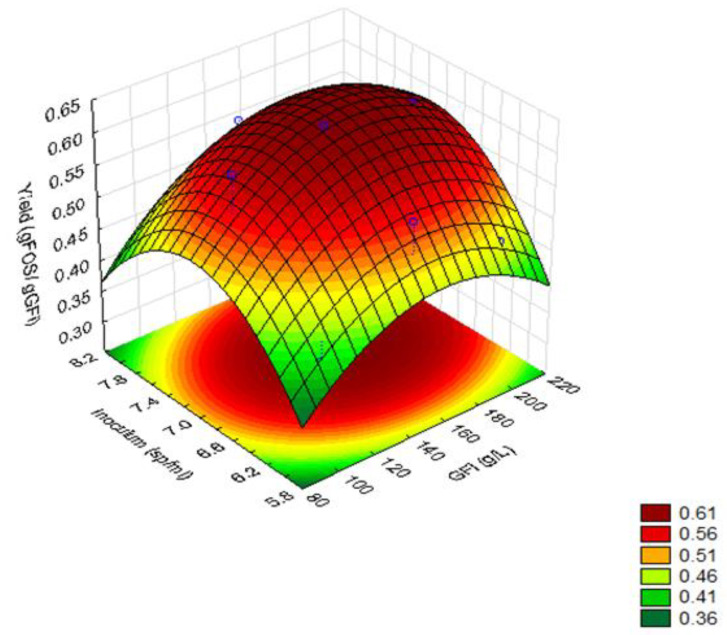
Response Surface plot of the effect of initial sucrose (GFi) concentration and inoculum concentration on fructooligosaccharides production yield (g_FOS_/g_GFi_).

Furthermore, with the response surface methodology, the values close to the midpoint of sucrose concentration and inoculum concentration favored a better yield in FOS production. The effects of the initial sucrose concentration and inoculum concentration on FOS yield are represented in Figure 2.

Based on the equation given by the model (Y = −4.58 − 0.97 × In^2^) and with the support of the statistical software STATISTICA 7.0, an estimation of the parameters that leads to maximum production of FOS was obtained. The model (R^2^ = 0.84) was statistically significant (*p* = 0.017) at a 95% confidence level (Table S2). Using 165.19 g/L of initial sucrose concentration and an inoculum concentration of 1 × 10^7^ sp/mL, the model predicted a yield of 0.63 g_FOS_/g_GFi_. The results obtained experimentally were in good agreement with the predicted values, as a yield of 0.64 ± 0.05 g_FOS_/g_GFi_ was obtained. 

**Figure 3:**
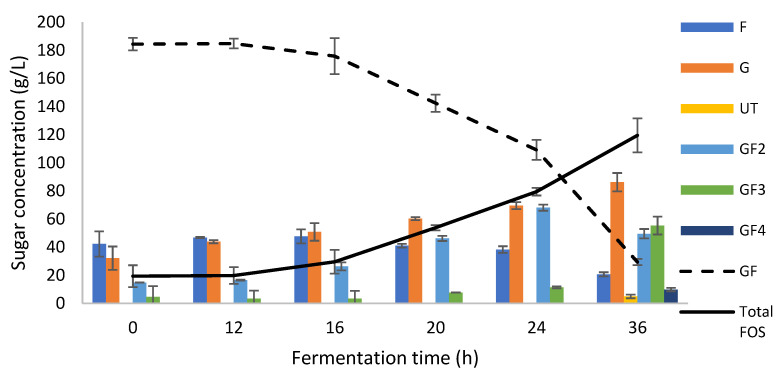
Fructooligosaccharides (FOS) production during fermentation under optimized conditions using AgMe media in shaking flasks. Fructose (F), glucose (G), sucrose (GF), unidentified compound, probably a trisaccharide (UT), kestose (GF2), nystose (GF3), 1-Fructofuranosyl nystose (GF4).

To validate the model, assays were carried out in triplicate in a flask. Experiments run under the estimated optimum operating conditions obtained a yield of 0.64 ± 0.05 g_FOS_/g_GFi_, which was slightly higher than that estimated by the model, with an FOS concentration of 119 ± 12 g/L at 36 h of fermentation (Figure 3). The FOS mixture consisted of 49.5 ± 3.4 g/L of GF_2_, 55 ± 6 g/L of GF_3_, 10 ± 1 g/L of GF_4,_ and 5 ± 1 g/L of a non-identified compound, most probably a saccharide. In the HPLC chromatograms, the unidentified compound appeared with a retention time different from the inulin-type FOS standards used and a retention time close to the GF_2_; thus, it is presumably a trisaccharide (Figure S2).

A reducing trisaccharide was also found in the FOS mixtures produced by other fungi, namely, *Aspergillus ibericus* and *Penicillium citreonigrum* [26,49,50]. After the analysis of its glycosidic linkage, three possible assignments were considered by the authors, namely, neokestose [Fru(β2 → 6)Glc(α1 ↔ β2)Fru], [Fru(β2 → 6)Glc(α1 ↔ α1)Glc], and theanderose [Glc(α1 → 6)Glc (α1 ↔ β2)Fru]. Other authors have reported the production of neo-FOS from sucrose by the fungi *Penicillium sizovae*, *Cladosporium cladosporioides,* and *Xanthophylomyces dendrorhous* [51,52,53]. The formation of neo-FOSs by these fungal strains can be attributed to invertases with fructosyltransferase activity produced by these fungi [54]. Thus, it is highly probable that the *A. oryzae* strain used in the present study also produces neokestose.

### 3.3. Bioreactor Trial

#### Bioreactor Trial with AgMe Media

After validating the optimal conditions at flask scale using AgMe media, and having observed good yields for the conditions assayed, experiments were scaled-up and carried out at a bioreactor scale using similar operational conditions. Trials in bioreactor showed slower FOS production as compared to fermentations at a flask level. In the bioreactor, the highest yield obtained during fermentation (0.60 g_FOS_/g_GFi_) was reached only at 108 h. The obtained yield was also lower than that obtained at a flask scale. A total amount of 103.03 g/L of FOS was produced. The lower performance may be associated with the different mechanical conditions of the Erlenmeyer and bioreactor, which influences the agitation and aeration of the medium. The high viscosity of the AgMe medium caused greater difficulty in achieving homogenization between the medium and biomass, generating precipitation inside the reactor. The high viscosity can lead to poor oxygen and mass transfer, delaying sucrose’s conversion into FOS. It is reported that fungal fermentation systems that suffer from high broth viscosity often lead to O_2_ mass-transfer limitation, which is associated with the broth viscosity itself and the biomass cell morphology [55]. Therefore, biomass formation in pellets is preferred since it can considerably decrease the broth viscosity. 

Fermentation carried out with Czapek-Dox media obtained faster sucrose conversion to FOS. In this case, a high concentration of sucrose along with low monosaccharides concentration promoted the synthesis of FOS. In addition, as the media homogenization through the bioreactor was better, fungal biomass grew in pellet form and was more dispersed in the media. Moreover, the microorganism growth was evident from the first hours of fermentation (observing a significant increase in the number of pellets in the media). The best FOS yield was obtained after 20 h of fermentation (0.612 ± 0.003 g g_FOS_/g_GFi_) with a production of 106.13 ± 11.26 g/L of total FOS composed mainly of GF_2_ (78.43 ± 10.21 g/L) and GF_3_ (27.66 ± 1.69 g/L). After this fermentation time, total FOS concentration began to decrease. At the bioreactor scale, FOS production yield with Czapek-Dox medium (0.61 ± 0.00 g_FOS_/g_GFi_) was similar to that obtained in AgMe medium, but at the flask level (0.60 g g_FOS_/g_GFi_). FOS production yield with AgMe media was the highest obtained yield (0.64 ± 0.05 g_FOS_/g_GFi_). Thus, the AgMe medium has proved excellent potential for the production of FOS. Nonetheless, improved conditions have to be tested in the bioreactor, e.g., using a different type of reactor as an airlift. 

The yields obtained in the present work using AgMe media were among the best results reported in FOS production in the literature. A FOS yield of 0.58 g_FOS_/g_GFi_ was reported by Sangeetha et al. using an *A. oryzae* in a two-stage continuous process [56]. A one-step bioprocess using *Aureobasidium pullulans* under optimized temperature and agitation speed reported a total FOS production yield of 0.64 g_FOS_/g_GFi_ after 51 h of fermentation [23]. Nobre et al. optimized temperature and initial pH conditions for FOS production by an *A. ibericus* at a flask level. A FOS yield of 0.53 g g_FOS_/g_GFi_ was obtained; however, when scaling up the process to a bioreactor level, the yield increased to 0.64 g_FOS_/g_GFi_ [18]. On the other hand, in the present work, a decrease in FOS yield was observed when scaling up the process from flask to a bioreactor level, from 0.64 g_FOS_/g_GFi_ to 0.60 g_FOS_/g_GFi_. Nevertheless, it is essential to highlight that AgMe is a media formulated from cheap sucrose alternatives, and similar FOS yields were obtained compared to defined synthetic media. 

Other non-synthetic media, such as the spent osmotic solutions, have been evaluated for FOS production in shaken flasks and bioreactors [57]. Yields of 0.37 g_FOS_/g_GFi_ and 0.34 g_FOS_/g_GFi_ have been reported for Andes berry, and Tamarillo fruit spent osmotic solutions using shaken flasks. At a bioreactor scale, yields were improved to 0.49 g_FOS_/g_GFi_ and 0.58 g_FOS_/g_GFi_, respectively. FOS yields achieved were lower than the yields obtained in this work with AgMe media (0.64 g_FOS_/g_GFi_). In terms of recent studies using molasses, most of them focus on media development for fungal growth or enzyme production and enzymatic synthesis of FOS, obtaining interesting results [38,42,58,59] suggesting molasses as a good low-cost alternative for enzyme production and now being corroborated by this study as an alternative for the production of FOS using whole cells in a single step fermentation.

FOS yields obtained by other authors using whole-cell fermentation process (including synthetic optimized media), under optimized conditions at flask level, were 0.53 g_FOS_/g_GFi_ for an *A. ibericus* [18], 0.56 ± 0.03 g_FOS_/g_GFi_ for a co-culture of *A. ibericus* and *Saccharomyces cerevisiae* [25] and 0.55 ± 0.00 g_FOS_/g_GFi_ for a *P. citroenigrum* [26]. It is essential to highlight that the yield obtained in this investigation with AgMe media (0.64 ± 0.05 g_FOS_/g_GFi_) was higher than the results reported by the other authors at the flask level. Still, all the processes were able to improve their yields when scaling up to bioreactor level (from 0.53, 0.55, and 0.55 to 0.64, 0.70, and 0.64 g_FOS_/g_GFi_, respectively [18,25,26]). Therefore, the next challenge using AgMe media will be to improve or even maintain the FOS yield obtained at flask level when scaling up to the bioreactor level, considering the physical challenges that may arise due to broth viscosity.

### 3.4. FOS Mixture Purification by Successive Fermentation with S. cerevisiae

A successive fermentation was carried out with the yeast *S. cerevisiae* to remove the residual sugars (glucose, fructose, and sucrose) from the FOS mixture generated during the production of FOS to improve FOS purity (% *w*/*w*) in the mixture. Two strains of *S. cerevisiae* were evaluated, namely, *S. cerevisiae 227* (S227) and *S. cerevisiae* 200 (S200). The results are discussed below.

#### 3.4.1. FOS Mixture Purification with *S. cerevisiae* 227

**Figure 4:**
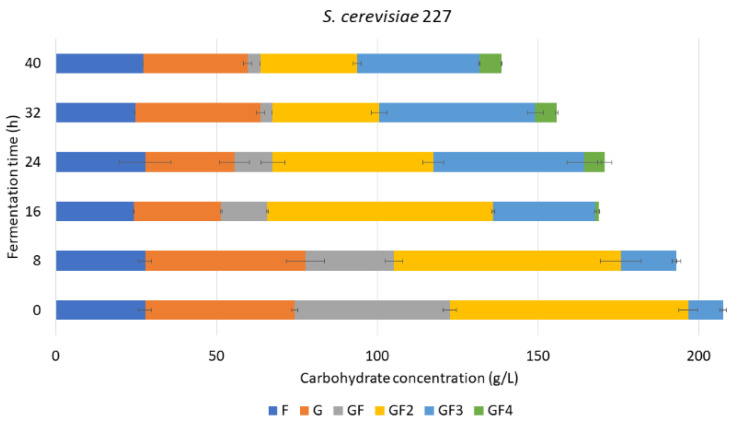
Carbohydrate concentration during successive fermentation with *Saccharomyces cerevisiae* 227 on AgMe fermentation mixture.

Successive fermentation successfully improved FOS% in the mixture from 39 ± 3% to 61 ± 1% (*w*/*w*) based on the amount of FOSs in relation to other carbohydrates in the mixture (after 16 h of fermentation) (Figure 4). Sucrose hydrolysis and FOS formation were observed during fermentation with the strain S227. At this point of the fermentation, it a decrease was observed in the amount of sucrose from 48.3 ± 2.1 g/L to 14.4 ± 0.3 g/L with a reduction in GF_2_ from 74.1 ± 2.9 g/L to 70.2 ± 0.4 g/L, an increase in the amount of GF_3_ from 10.8 ± 1.0 to 31.8 ± 0.3 g/L, with the appearance of GF_4_ in a concentration of 1.12 ± 0.01 g/L (Figure S3). Although the fermentation extract was filtered to eliminate the biomass of the fungi before starting the second fermentation, some extracellular enzymes may have maintained their FOS synthesis activity and sucrose hydrolysis [24]. Nevertheless, the amount of glucose did not increase during the transfructosylation, meaning that it is being consumed by the *Saccharomyces*.

#### 3.4.2. FOS Mixture Purification with *S. cerevisiae* 200

**Figure 5:**
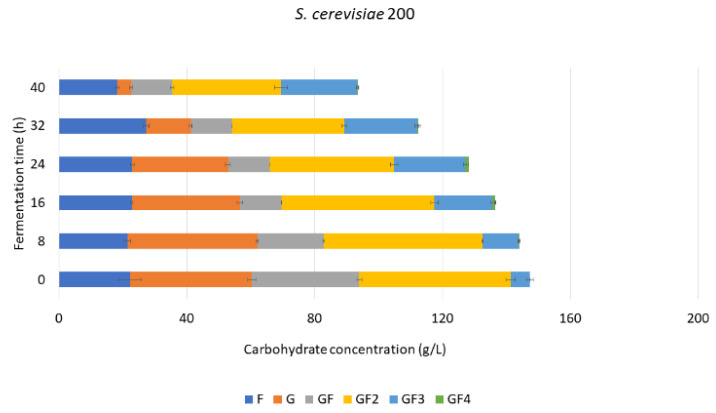
Carbohydrate concentration during successive fermentation with *Saccharomyces cerevisiae* 200 on AgMe fermentation mixture.

During the fermentation with the strain S200, sucrose was gradually depleted, reaching its minimum concentration (12.8 ± 0.6 g/L) at 40 h fermentation (Figure 5), which was also the time at which the highest percentage of FOS was reached, 62 ± 3% (*w*/*w*) compared to 39 ± 3% of FOS in the mixture before successive fermentation. FOS synthesis was observed during the fermentation. While the concentration of GF_2_ decreased during the fermentation, from 47.3 ± 1.4 g/L to 34.0 ± 2.0 g/L, the concentration of GF_3_ increased from 6.05 ± 1.02 to 23.9 ± 0.4 g/L, and GF_4_ started being synthesized in small amounts after 16 h fermentation (0.83 ± 0.07 g/L), and its final concentration decreased to 0.19 ± 0.03 g/L after 40 h fermentation (Figure S4).

This FOS synthesis activity could be associated either with a possible FOS synthesis ability of both *Saccharomyces* strains used or by remaining *A. oryzae* enzymes in the fermentation medium, which could maintain their synthesis activity and the hydrolysis of sucrose. However, more FOS production was observed during fermentation with the S227 strain. A similar case was reported by Nobre et al. [24], where FOS synthesis was observed during a successive fermentation when fungi biomass was previously removed. The author attributed the FOS synthesis activity to the possible remaining enzymes from the fungi. In another work, a strain of *S. cerevisiae* among other yeasts and fungus was evaluated for its FOS-producing capacity. *S. cerevisiae* exhibited transfructosylating and hydrolytic activity and was able to produce FOSs in low amounts [60].

The mechanism of action of the yeast *Saccharomyces cerevisiae* towards sucrose and mixtures for sucrose, glucose, and fructose have been described. The yeast can hydrolyze sucrose since it is reported that it has genes to produce invertases (SUC genes) [61]. *Saccharomyces* produce extracellular invertases that perform sucrose hydrolysis outside the cell generating monosaccharides glucose and fructose, then these monosaccharides are introduced by facilitated diffusion and become available to be phosphorylated as the first step of the Embden–Meyerhof–Parnas glycolytic pathway [62].

**Figure 6:**
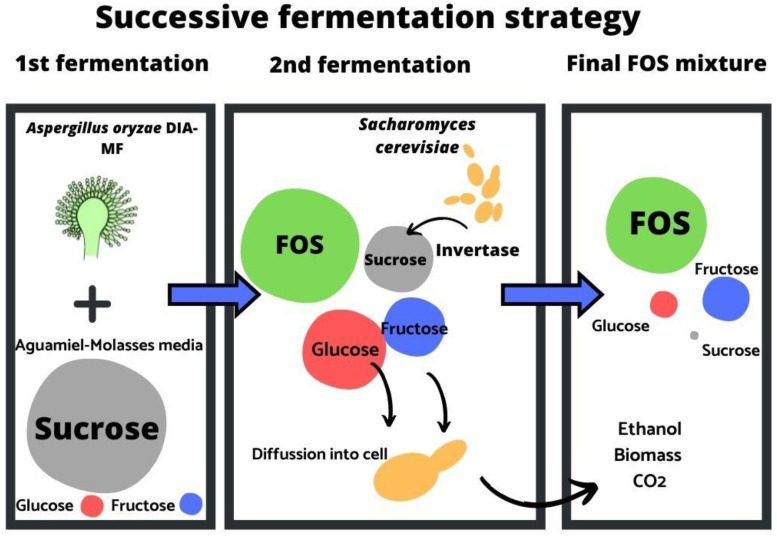
Schematic representation of successive fermentation. First fermentation: fructooligosaccharides (FOS) synthesis by *Aspergillus oryzae* DIA-MF in Aguamiel-Molasses (AgMe) media. Second fermentation: *Saccharomyces cerevisiae* acts on the FOS mixture reducing the content of mono- and disaccharides.

Glucose and sucrose concentrations play key roles in gene expression and carbohydrate consumption. As the yeast cells want to work more efficiently, the SUC gene is not expressed unless there is a higher sucrose concentration than glucose; otherwise, sugars other than glucose are consumed after the depletion of glucose. FOSs also influence the production of invertases by yeast. For that reason, the yeast begins to hydrolyze sucrose, releasing more glucose and sucrose, which are then consumed consecutively being fructose the least in terms of energetic efficiency [63,64]. A schematic diagram of the successive fermentation is represented in Figure 6.

A higher FOS% was obtained in the fermentation mixtures treated with the successive fermentation with strains S227 and S200. However, the strain S227 showed more efficiency, reaching 61.04 ± 0.64% of FOS in 16 h of fermentation in comparison with S200, which reached the highest FOS% (62.12 ± 2.57) after 40 h of fermentation time. 

In terms of purity, other authors have also performed yeast treatment to purify oligosaccharide mixtures. In a similar study, Hernandez et al. [65] purified the commercial product Vivinal-GOS^®^ (consisting of galactooligosaccharides (GOS)) with yeast treatment with *S. cerevisiae* for 24 h. Maximum GOS purity was achieved at 10 h, increasing GOS in the mixture from 38.6% to 50.6%. Other authors have reported the successive fermentation strategy for FOS production. The highest yield obtained was 84.4% of FOS purity, which is considerably higher than that obtained in this work [24]. Still, it is essential to note that in that process, the medium composition was optimized to ensure yeast growth to allow for an easier purification of the FOS mixture. Therefore, further optimization of the purification process of the FOS mixtures obtained from AgMe media may ensure increased FOS purity. 

## 4. Conclusions

This work shows that it is possible to use Aguamiel and molasses for media formulation and as sucrose alternatives to obtain a high yield in FOS production (0.61 ± 0.06 g_FOS_/g_GFi_). By Response Surface Methodology, the initial concentration of sucrose in the AgMe medium (165.2 g/L) and the inoculum concentration (1 × 10^7^ sp/mL) were defined by the experimental design, allowing to increase the production of total FOS from 46.7 to 119.5 g/L and the yield from 0.61 ± 0.06 to 0.64 ± 0.05 g_FOS_/g_GFi_. 

The production of a new compound, probably the trisaccharide neo-kestose, was identified during fermentation with the AgMe medium, which, to our knowledge, has never been reported to the *A. oryzae* strain. 

FOS mixtures were purified through successive fermentation with *S. cerevisiae* S277, increasing FOS purity in mixtures from 39 ± 3% up to 61.04 ± 0.64% and resulting in a product with reduced mono- and disaccharides. 

The fermentation process herein optimized showed that it is possible to produce high-value prebiotic FOSs at a high-yield using low-cost substrates and agro-industrial by-products.

## Figures and Tables

**Table 1:** The central composite design was applied to experimental variables: initial sucrose concentration (GFi) and inoculum concentration.

Experimental Run	GFi (g/L)	Inoculum 10^(Spore/mL)
1	100 (−1)	6 (−1)
2	100 (−1)	8 (+1)
3	200 (+1)	6 (−1)
4	200 (+1)	8 (+1)
5	100 (−1)	7 (0)
6	200 (+1)	7 (0)
7	150 (0)	6 (−1)
8	150 (0)	8 (+1)
9	150 (0)	7 (0)
10	150 (0)	7 (0)
11	150 (0)	7 (0)

**Table 2:** Fructooligosaccharides (FOS) maximum production (Max. FOS), yield, and purity achieved during fermentation with the different media evaluated at optimal fermentation time.

Media	Gfi (g/L)	Max. FOS (g/L)	Biomass (g/L)	Time (h)	Yield (g_FOS_/g_GFi_)	Productivity (g_FOS_/L∗h)	Purity%
Aguamiel	82.34 ± 12.30	45.93 ± 1.13	0.31 ± 0.06	32	0.55 ± 0.01	1.44 ± 0.03	37.23 ± 0.91
AgMe	76.27 ± 9.18	46.73 ± 0.31	2.14 ± 0.39	16	0.61 ± 0.06	2.92 ± 0.03	39.99 ± 0.26
Me	168.62 ± 18.59	101.63 ± 6.48	4.31 ± 0.20	24	0.60 ± 0.05	4.23 ± 0.33	38.39 ± 0.95

Gfi: Initial sucrose.

**Table 3:** Fructooligosaccharides production yields obtained from the experimental runs and predicted values of the central composite design.

	Yield (g_FOS_/g_GFi_)	
Experimental Run	Experimental Value	Predicted Value
1	0.41	0.48
2	0.45	0.42
3	0.47	0.51
4	0.55	0.53
5	0.61	0.55
6	0.61	0.59
7	0.56	0.51
8	0.56	0.56
9	0.60	0.63
10	0.61	0.63
11	0.63	0.62

## Data Availability

Data is contained within the article and Supplementary Material.

## Supplementary Materials

The following supporting information can be downloaded at: https://www.mdpi.com/article/10.3390/foods11121786/s1, Figure S1: Schematic representation of fructooligosaccharides (FOS) structures obtained by synthesis during fermentation with strain *Aspergillus oryzae* DIA-MF. DP: degree of polymerization; 1-FN: 1-Fructofuranosyl nystose. title; Figure S2: Chromatograms obtained by HPLC for: a mixture of standard fructooligosaccharides (FOS) (Black line) and for a fermentation broth sample obtained from the fermentation of Aguamiel and Molasses (AgMe) with *Aspergillus oryzae* (Orange line). Figure S3: Chromatograms obtained by HPLC for: for a fermentation broth sample obtained from the fermentation of Aguamiel and Molasses (AgMe) with *Aspergillus oryzae* before (black line) and after (pink line) inoculation of *Saccharomyces cerevisiae 227* (S227). Figure S4: Chromatograms obtained by HPLC for: for a fermentation broth sample obtained from the fermentation of Aguamiel and Molasses (AgMe) with *Aspergillus oryzae* before (black line) and after (green line) inoculation of *Saccharomyces cerevisiae 200* (S200). Table S1: Sugar concentration obtained on the aguamiel from Agave salmiana and on the sugar cane molasses used as raw materials for media formulation. Table S2: Statistical analysis of the full Central Composite Design with two factors used for the optimization of fructooligosaccharides production.
