# Supplementary material for: Successive Fermentation of Aguamiel and Molasses by Aspergillus oryzae and Saccharomyces cerevisiae to Obtain High Purity Fructooligosaccharides
Source: Foods. 2022 Jun 17;11(12):1786. doi: 10.3390/foods11121786 (PMC9222578; doi:10.3390/foods11121786)
Supplement: Supplementary file 1 [file foods-11-01786-s001.zip › foods-1726362-supplementary.pdf]

Supplementary Material

Successive Fermentation of Aguamiel and Molasses by *Aspergillus oryzae* and *Saccharomyces cerevisiae* to Obtain High Purity Fructooligosaccharide

Orlando de la Rosa [1], Adriana Carolina Flores-Gallegos [1], Diana Muñíz-Márquez [2], Juan C. Contreras-Esquivel [1], José A. Teixeira [3,4], Clarisse Nobre [3,4,*] and Cristóbal N. Aguilar [1,*]

[1] Bioprocesses & Bioproducts Research Group, Food Research Department, School of Chemistry, Univer-sidad Autónoma de Coahuila, Saltillo 25280, Mexico; orlandorosaflores@uadec.edu.mx (O.d.l.R.); carolinaflores@uadec.edu.mx (A.C.F.-G.); carlos.contreras@uadec.edu.mx (J.C.C.-E.)
[2] Tecnológico Nacional de México, Instituto Tecnológico de Ciudad Valles, Ciudad Valles 79010, Mexico; diana.marquez@tecvalles.mx
[3] Centre of Biological Engineering, Campus de Gualtar, University of Minho, 4710-057 Braga, Portugal; jateixeira@deb.uminho.pt
[4] LABBELS—Associate Laboratory, 4710-057 Braga, Portugal
* Correspondence: clarissenobre@deb.uminho.pt (C.N.); cristobal.aguilar@uadec.edu.mx (C.N.A.)

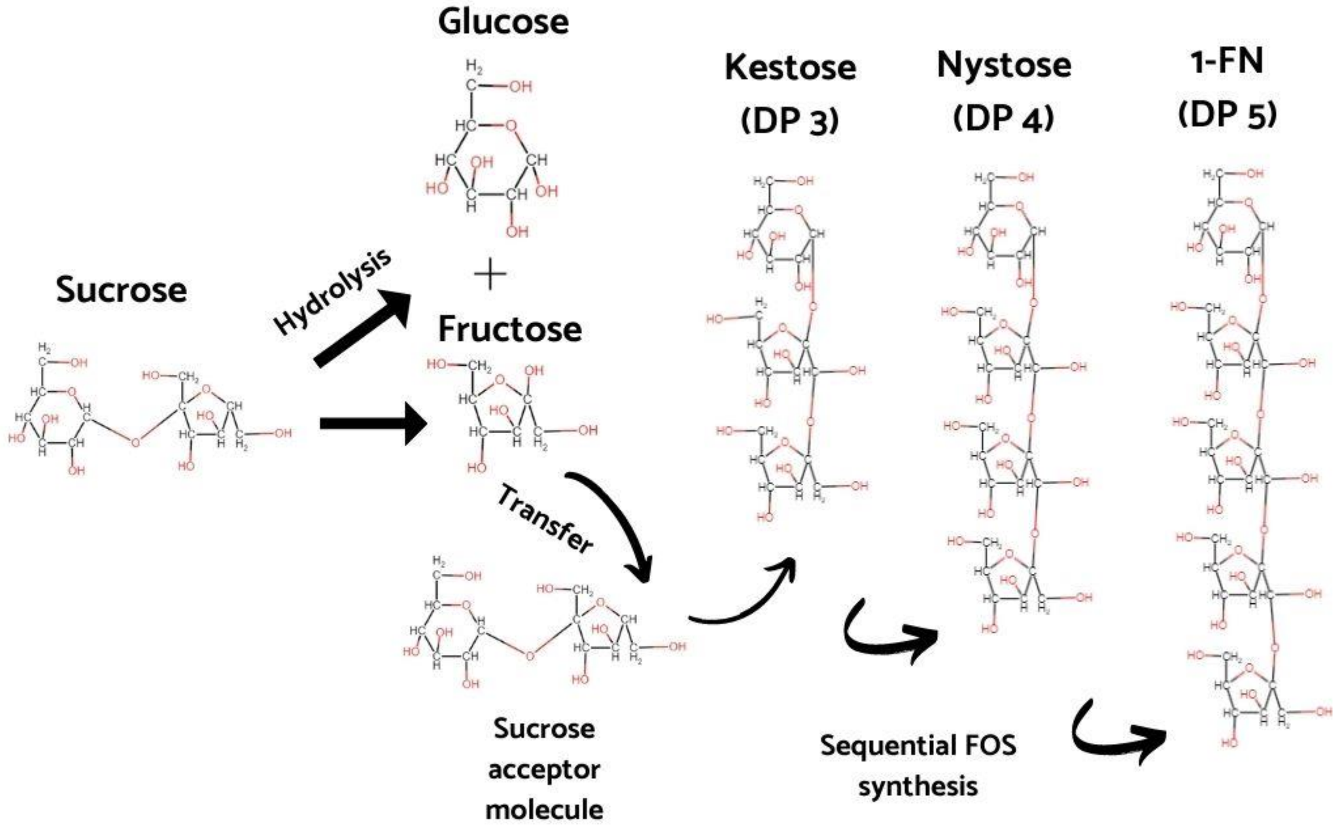

Figure S1. Schematic representation of fructooligosaccharides (FOS) structures obtained by synthesis during fermentation with strain *Aspergillus oryzae* DIA-MF. DP: degree of polymerization; 1-FN: 1-Fructofuranosyl nystose.

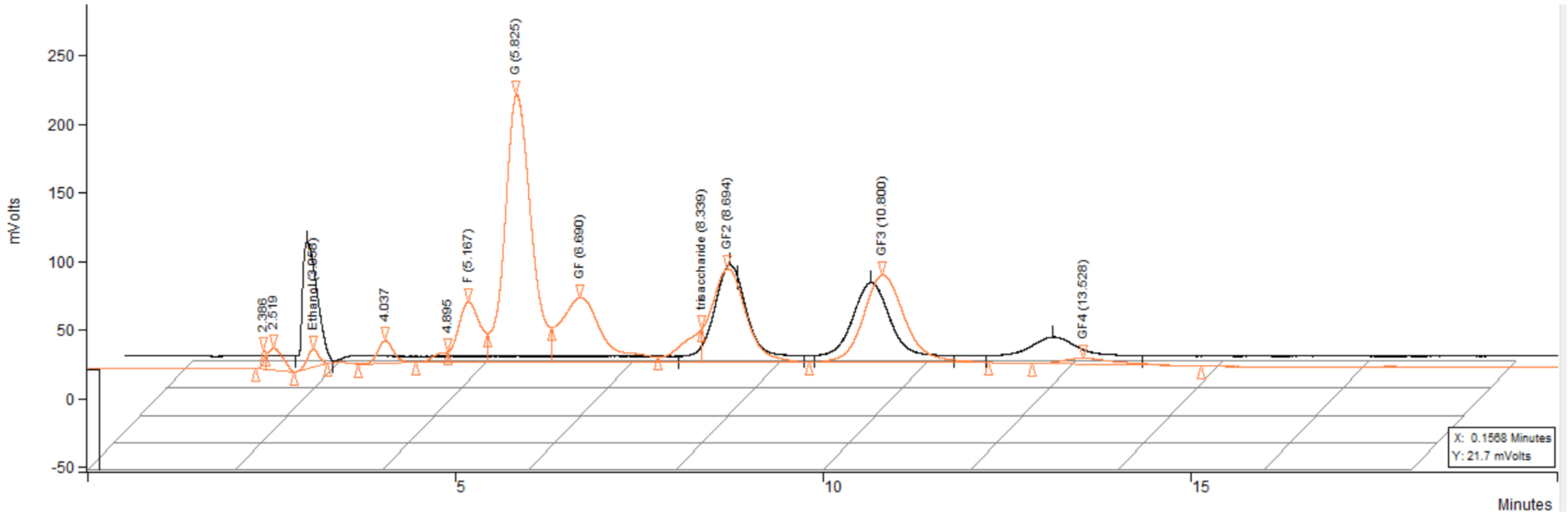

Figure S2. Chromatograms obtained by HPLC for: a mixture of standard fructooligosaccharides (FOS) (Black line) and for a fermentation broth sample obtained from the fermentation of Aguamiel and Molasses (AgMe) with *Aspergillus oryzae* (Orange line). Fructose (F), glucose (G), sucrose (GF), unidentified compound - possibly a trisaccharide, kestose (GF2), nystose (GF3), 1-Fructofuranosyl nystose (GF4).

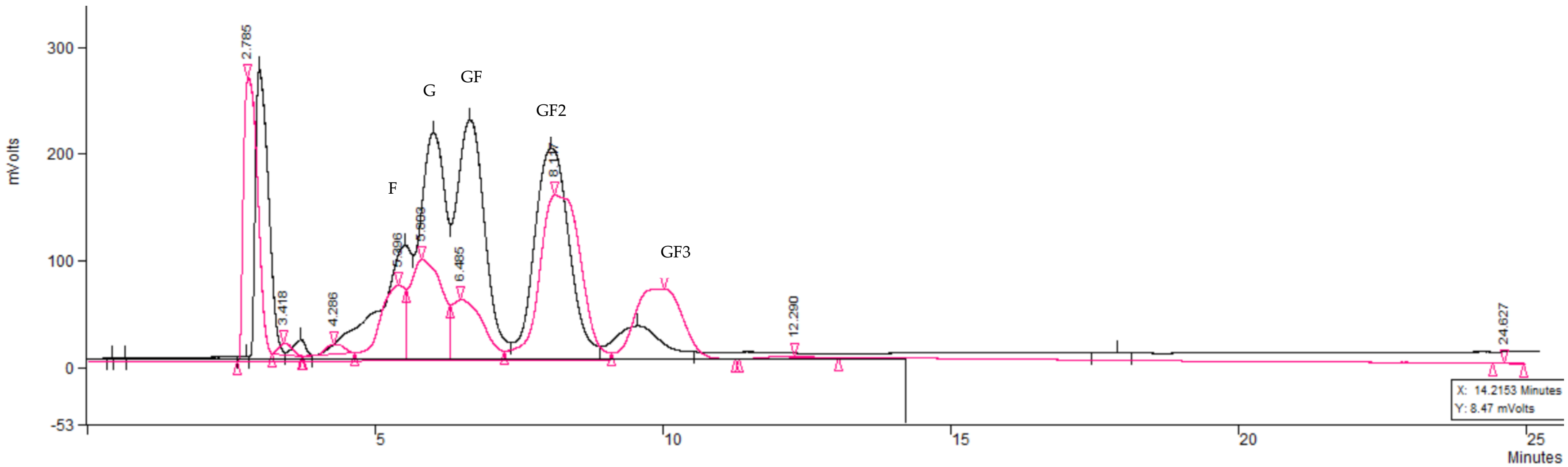

Figure S3. Chromatograms obtained by HPLC for: for a fermentation broth sample obtained from the fermentation of Aguamiel and Molasses (AgMe) with *Aspergillus oryzae* before (black line) and after (pink line) inoculation of *Saccharomyces cerevisiae 227* (S227). Fructose (F), glucose (G), sucrose (GF), kestose (GF2), nystose (GF3).

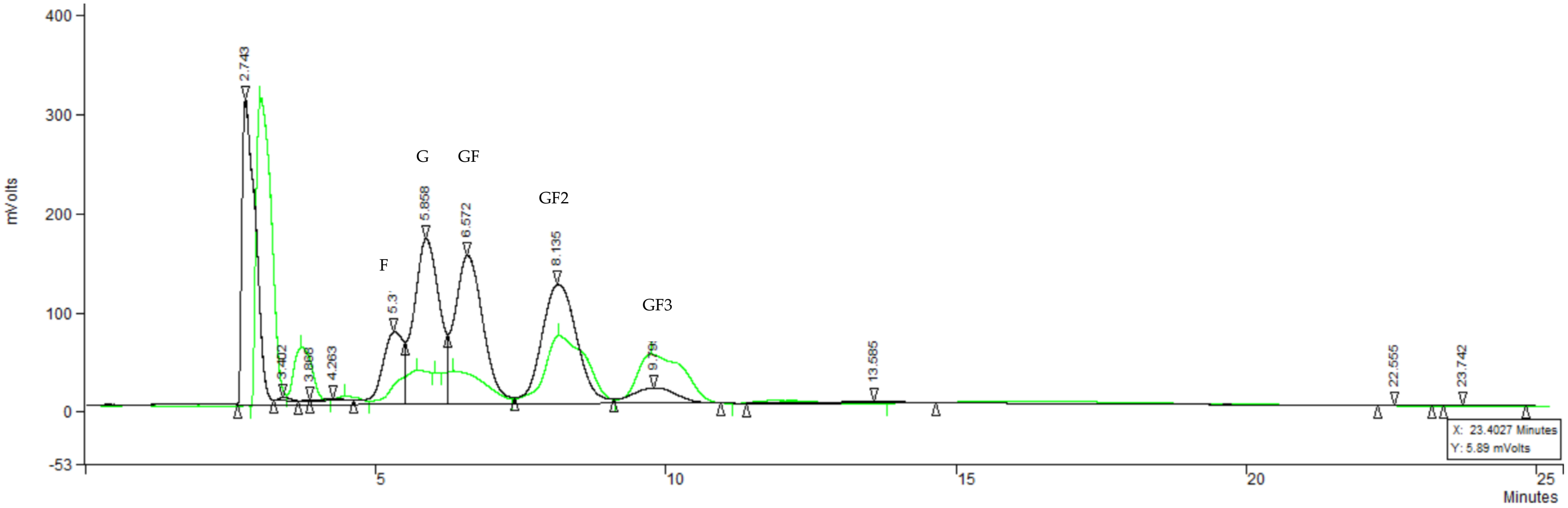

Figure S4. Chromatograms obtained by HPLC for: for a fermentation broth sample obtained from the fermentation of Aguamiel and Molasses (AgMe) with *Aspergillus oryzae* before (black line) and after (green line) inoculation of *Saccharomyces cerevisiae 200* (S200). Fructose (F), glucose (G), sucrose (GF), kestose (GF2), nystose (GF3).

Table S1. Sugar concentration obtained on the aguamiel from *Agave salmiana* and on the sugar cane molasses used as raw materials for media formulation.

Raw material	Fructose (g/L)	Glucose (g/L)	Sucrose (g/L)
Aguamiel	4.17 ±0.21	4.33 ±0.39	98.56 ±9.77
Molasses	96.10 ±9.56	71.35 ±6.20	441.41 ±42.02

Table S2. Statistical analysis of the full Central Composite Design with two factors used for the optimization of fructooligosaccharides production.

**Variables and interactions**	**Estimated effects**	**Standard Error**	**t-value**	***p***
Mean/Interc	-4.580	1.307	-3.504	0.017*
(1) GFi (L)	0.005	0.004	1.165	0.296
GFi (Q)	0.000	0.000	-1.855	0.123
(2) Inoc (L)	1.348	0.371	3.631	0.015*
Inoc (Q)	-0.097	0.026	-3.711	0.013*
1L by 2L	0.000	0.000	0.474	0.656

GFi: Initial sucrose concentration, Inoc: Inoculum concentration

*Significant influence at 95% confidence level.
